# A Systematic Review of International Clinical Guidelines for Rehabilitation of People With Neurological Conditions: What Recommendations Are Made for Upper Limb Assessment?

**DOI:** 10.3389/fneur.2019.00567

**Published:** 2019-06-25

**Authors:** Jane Burridge, Margit Alt Murphy, Jaap Buurke, Peter Feys, Thierry Keller, Verena Klamroth-Marganska, Ilse Lamers, Lauren McNicholas, Gerdienke Prange, Ina Tarkka, Annick Timmermans, Ann-Marie Hughes

**Affiliations:** ^1^Faculty of Environmental and Life Sciences, University of Southampton, Southampton, United Kingdom; ^2^Institute of Neuroscience and Physiology, Rehabilitation Medicine, Sahlgrenska Academy, University of Gothenburg, Gothenburg, Sweden; ^3^Roessingh Research and Development, Enschede, Netherlands; ^4^Faculty of Electrical Engineering, Mathematics and Computer Science, University of Twente, Enschede, Netherlands; ^5^REVAL Rehabilitation Research Center, Faculty of Rehabilitation Sciences, Hasselt University, Hasselt, Belgium; ^6^Tecnalia Research & Innovation, San Sebastian, Spain; ^7^Zurich University of Applied Science (ZHAW), Winterthur, Switzerland; ^8^Faculty of Engineering Technology, University of Twente, Enschede, Netherlands; ^9^Faculty of Sport and Health Sciences, University of Jyväskylá, Jyväskylä, Finland

**Keywords:** practice guidelines, neurological conditions, upper limb, outcome and process assessment, systematic review, guidelines, impairment, activity

## Abstract

**Background:** Upper limb impairment is a common problem for people with neurological disabilities, affecting activity, performance, quality of life, and independence. Accurate, timely assessments are required for effective rehabilitation, and development of novel interventions. International consensus on upper limb assessment is needed to make research findings more meaningful, provide a benchmark for quality in clinical practice, more cost-effective neurorehabilitation and improved outcomes for neurological patients undergoing rehabilitation.

**Aim:** To conduct a systematic review, as part of the output of a European COST Action, to identify what recommendations are made for upper limb assessment.

**Methods:** We systematically reviewed published guidance on measures and protocols for assessment of upper limb function in neurological rehabilitation via electronic databases from January 2007–December 2017. Additional records were then identified through other sources. Records were selected for inclusion based on scanning of titles, abstracts and full text by two authors working independently, and a third author if there was disagreement. Records were included if they referred to “rehabilitation” and “assessment” or “measurement”. Reasons for exclusion were documented.

**Results:** From the initial 552 records identified (after duplicates were removed), 34 satisfied our criteria for inclusion, and only six recommended specific outcome measures and /or protocols. Records were divided into National Guidelines and other practice guidelines published in peer reviewed Journals. There was agreement that assessment is critical, should be conducted early and at regular intervals and that there is a need for standardized measures. Assessments should be conducted by a healthcare professional trained in using the measure and should encompass body function and structure, activity and participation.

**Conclusions:** We present a comprehensive, critical, and original summary of current recommendations. Defining a core set of measures and agreed protocols requires international consensus between experts representing the diverse and multi-disciplinary field of neurorehabilitation including clinical researchers and practitioners, rehabilitation technology researchers, and commercial developers. Current lack of guidance may hold-back progress in understanding function and recovery. Together with a Delphi consensus study and an overview of systematic reviews of outcome measures it will contribute to the development of international guidelines for upper limb assessment in neurological conditions.

## Introduction

Worldwide prevalence of stroke in 2010 was 33 million, with 16.9 million people having a first stroke, of which 795,000 were American and 1.1 million European (1). It has been estimated that approximately one third of people fail to regain upper limb capacity, despite receiving therapy (2). This has important implications for both individuals and the wider society as reduced upper limb function is associated with dependence and poor quality of life for both patients and carers (3–5) and impacts on national economies (6).

While stroke has the highest prevalence, other neurological conditions such as Multiple Sclerosis (MS), Spinal Cord Injury (SCI), and Traumatic Brian Injury, have a significant incidence and there are often similarities in presentation, and treatment and therefore assessment. The worldwide incidence of SCI is 40–80 cases per million population and the estimated European mean annual rate of MS incidence is 4.3 cases per 100,000 (7). Recently, Kister et al. (8) reported that 60% of people with MS have impaired hand function. The impact of upper limb dysfunction on ADL is higher than in stroke, as both sides are often affected (9). Although dysfunction after SCI depends on level of injury, upper limb function is consistently cited as a health priority. The incidence rate of TBI in Europe is about 235 per 100,000 population (10). Outcome data among European countries are very heterogeneous. From the US however, it is known that about 1.1% of the population suffer a TBI resulting in long term disability (11).

### Rationale

Providing evidence-based and cost-effective upper limb rehabilitation is a priority for patients and healthcare services and is increasingly important because of the growth in new technology-based interventions designed to augment conventional occupational therapy and physical therapy. Outcome data are key to delivering best practice and identifying which interventions are effective. To design trials that will deliver unequivocal results, so that useful, and only useful interventions can be translated into clinical practice and delivered optimally, we need to understand the complexity and interaction between patient and intervention. To do that requires a large amount of comparable data—i.e., data generated from an agreed small set of valid outcome measures (OM) using agreed protocols. By standardizing OM and protocols, aggregated data can be mined to generate a better understanding of what interventions are effective, at what dose, when, with whom and in what setting they should be used. This will enable clinicians to make better informed decisions and thus improve patient outcomes. Agreed, widely used, valid and practical OMs and assessment protocols are important in research into and treatment of all neurological conditions, but may be particularly important in conditions where incidence is lower and therefore data sets smaller.

Guidelines on best practice aim to improve treatment standards, including rehabilitation, and directing future research. And, as we argue above, OMs are key to achieving that goal. It would seem reasonable therefore that clinical guidelines would be a source of guidance on selection of OMs and protocols for their use. In this study, we have therefore systematically reviewed recent and current guidelines on stroke, MS, SCI, and TBI. We have excluded all other neurological disabilities such as Parkinson's Disease and cerebellar ataxia as the assessment protocols and tools for these conditions are very different. We have extracted recommendations on assessment in terms of outcome measures (OM), frequency of assessment and who should conduct assessments, when and with what purpose.

### Objectives

This study is one of three components in the development of European Guidelines on assessment of the upper limb in neurological conditions. Two studies have already been published: A Delphi study which reported the views of experts (12) and an overview of systematic reviews of OMs (13). The project was driven by a realization that progress in upper limb neurological rehabilitation research and consequently improvement in quality of care was hampered by the absence of consensus on OMs and protocols for assessment. To conduct effective metanalysis requires multiple clinical studies to use the same measures using comparable protocols, and for the same OMs to be used in clinical practice. Practice guidelines are an obvious source of information on useful measures and protocols for assessment. The objective of this study was therefore to explore published and web-based guidance and to extract and synthesize recommendations on assessment measures and protocols for assessment of upper limb function for people with neurological conditions.

### Research Question

Our research question was: What recommendations are made by international clinical guidelines for the assessment of the upper limb in neurological conditions?

## Methods

### Study Design and Search Strategy

Published studies were identified through Pubmed and Evidence Search databases (MEDLINE in Ovid, Embase, CINAHL, AMED, Web of Science, PEDro and Google Scholar) for the period from January 2007 to December 2017. The search strategy comprised the following medical subject heading (MeSH) terms: *stroke, multiple sclerosis, spinal cord injuries* and *neurological rehabilitation* with filters for *guidelines, recommendations, practice guidelines* and *consensus development conference*. The search was as follows *(((((“Stroke”[Mesh]) OR “Multiple Sclerosis”[Mesh]) OR “Spinal Cord Injuries”[Mesh]) OR “Traumatic Brain Injury”[Mesh]) OR “Neurological Rehabilitation”[Mesh])) AND (((Practice Guideline[pt] OR Recommendation OR Guideline[pt] OR Consensus Development Conference[pt])) AND (“2007/01/01”[PDat]: “2017/12/31”[PDat]))*. Using the search engine Google, applying the terms “[nation],” guideline, “stroke,” members of the COST action searched for their National Stroke Guidelines in their respective languages: UK, Netherlands, Italy, Spain, Germany, Switzerland, Sweden and Estonia. Using the same terms, we also searched, in English for any other National Guidelines from any country for stroke, SCI, MS, TBI or Neurological Conditions. Additional records were also identified through other sources, especially references from the retrieved records.

### Systematic Review Protocol and Data Extraction

Two review authors (JB and AH) independently screened references for relevance based on their abstract, and methodological quality, where there were any disagreements the wider group were consulted. Records were only included in the review if they referred to upper limb “assessment” or “measurement” and “physical rehabilitation” of “neurological disorders” and were either a “National Guideline” or either “practice guideline” or “recommendations” published in a peer-reviewed Journal. Additional studies were identified from references within the records and, where they satisfied these criteria were included in the review. Although our interest was primarily in upper limb assessment, the guideline literature usually encompassed the broad topic of assessment, i.e., both upper and lower limb, activities of daily living and impact on quality of life. Such articles were screened, but only included for further review when guidelines on upper limb assessment were included. We did not use a standard tool to assess quality. Records that satisfied the criteria for inclusion were then categorized by two independent authors (AH and JB) into: National guideline; other practice guidelines or recommendations published in peer-reviewed journals or web-based resources and then by condition into: stroke; multiple sclerosis (MS); Spinal cord injury (SCI), traumatic brain injury (TBI) or “other neurological conditions.” Each record was then reviewed (LM, JB and AH). Data were then extracted from each record and tabulated.

### Data Analysis

Based on the review a classification structure (see below) was designed to reflect the relevant areas in which recommendations were made.

Classification structure:

1. Why assessment is important
2. When during the rehabilitation should assessment be conducted
3. Clinical Utility—who should conduct the assessment
4. Single vs. multiple OMs within the ICF Framework
5. Assessment of body function and structures (impairment) and activity
6. Assessment of Activities of Daily Living (ADL) and participation
7. Psychometric properties and appropriateness of OMs
8. Self-Efficacy and goal orientated measures—assessment integrated into therapy.

## Results

**Figure 1:**
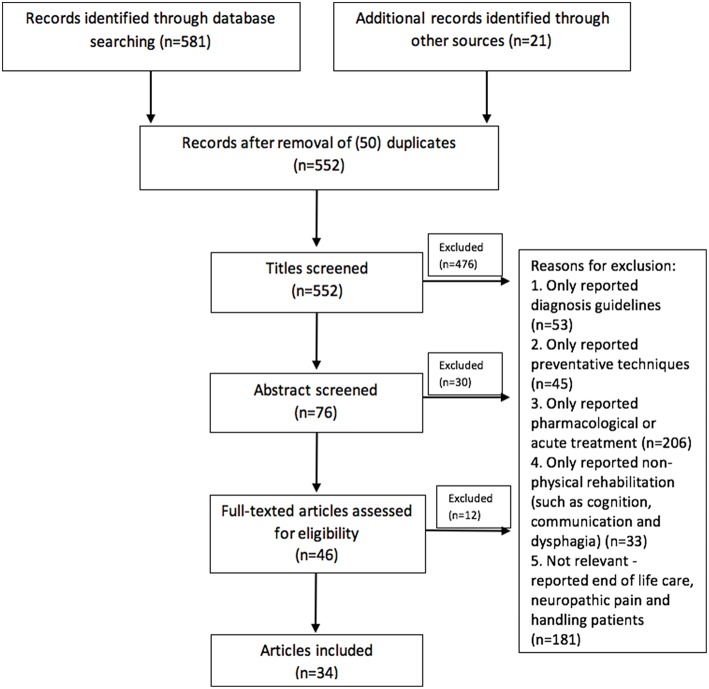
Flow diagram of the studies retrieved for the review.

The records retrieved for the review and the results of the selection process are shown in the flow diagram (Figure 1).

### Study Selection Characteristics

Our primary aim was to review and synthesize recommendations for the selection and use of upper limb OMs (both conventional and technology-based) in neurorehabilitation. Our search identified no records that focussed exclusively on the UL and the majority made only brief reference to either assessment or measurement tools (14–18). Where reference was made to measurement there was explicit consensus that measures should follow the World Health Organization (WHO) International Classification of Function (ICF) framework (19, 20).

### Synthesized Findings

Of the 34 publications included in the review only six (two National Guidelines)

recommended specific measures of body function and structures, activity and participation (14, 15, 17, 18, 21, 22). Seven recommended global scales but gave no specific measures for the upper limb (23–28). Most National Guidelines focussed on service delivery. Some acknowledged that standardized OMs are required for effective neurorehabilitation, without reference to specific tools or how they should be chosen. The need for OMs that encompass all domains of the ICF was agreed.

Nine publications referred to the importance of global or upper limb assessments being conducted by appropriately trained or qualified healthcare professionals (HCP) (22, 29–36). Protocols for and timing of assessment was only included in four records (17, 21, 22, 37). In total, reviews identified 47 different global and upper limb specific OMs, but only one referred to effectiveness, validity or reliability of the recommended measures (17).

Fourteen National Guidelines were included in the review (Table 1) from the following countries: The Netherlands, Sweden, UK (4), Scotland (2), Estonia, South Africa, Singapore, Australia, New Zealand and the USA. National guidelines were condition specific: 11 stroke, 1 brain injury, 1 SCI and 1 MS. National Guidelines provided the most comprehensive and broad recommendations. All National stroke guidelines except the South African (33) and Swedish (55) make some reference to assessment, but in almost all cases it was brief, non-specific and not related either to rehabilitation or the UL. There were two exceptions to this.

**Table 1:** Summary of the National Guideline records included in the review.

**Record**	**Year**	**Summary of recommendations**	**Recommended measures**
Australian Stroke Foundation (38). (Stroke)	2017	Use of valid measures; assessment made by trained clinicians. No reference to physical assessment of the upper limb	None
Winsteinet al. (14) (Stroke)	2016	Recommends a single assessment used throughout the course of stroke recovery	Computerized questionnaire: “*Activity Measure for Post-Acute Care”*; dynamometer (grip strength) (39); electro-goniometer (range of motion) (40) and Frey filaments (tactile sensory deficits) (41). Fugl-Meyer (42) and Box and Block Test (43)
Royal College of Physicians (16) (Stroke)	2016	Use of the WHO ICF and instruments appropriate to the intervention. Clinicians should be trained in the use of measurement scales; set agreed goals (including patient and carers)	None
Veerbeek et al. Dutch Guidelines (17) (Stroke)	2014	Measures that are valid, reliable, responsive and feasible within each ICF domain. Use for diagnosis, clinical decision-making, to predict recovery, and assess progress. Measure at predefined times to monitor recovery e.g., within one week of admission and discharge (or when transferring care) end of the 1st week, 3rd and 6th month post-stroke. Consider measures before each multidisciplinary meeting.	Motricity Index (44); Fugl-Meyer (FMA UE) (42) Frenchay Arm Test [FAT]) (45), Action Research Arm Test (ARAT) (46) and Nine Hole Peg Test (NHPT) (43). MAS (47); Nottingham Extended ADL (48); Global measures: SSQoL (49); Barthel Index (50), NIHSS (51)
NICE, Multiple Sclerosis (32). (MS)	2014	No reference to upper limb problems. Assessment should be conducted by a “healthcare professional with appropriate expertise in rehabilitation and MS”.	None
SIGN. Guideline 130 Brain injury rehabilitation in adults (26) (TBI)	2013	Brief reference to assessment and OM: “A range of tools can assist in the assessment and setting of goals”; no specific recommendations on measures or timing.	COPM (52), FIM/FAM (53), Barthel Index (50).
NICE. Stroke rehabilitation in adults - NICE guideline (28) (Stroke)	2013	Screen for impairment, activity limitations, participation restrictions, and environmental factors to direct treatment on admission and on transfer from hospital to community. Standardized valid and reliable screening instruments should be used by HCPs who have appropriate skills and training. Wrist and hand splints should be assessed and fitted by trained HCPs. In research, the primary outcome measure should be improvement in function, with secondary outcomes assessing impairment, function, and quality of life.	NIHSS (51); Barthel Index (50)
NSCISB. The National Spinal Cord Injury Strategy Board (54). (SCI)	2012	Only reference to rehabilitation is passive movement to maintain joint range with no reference to assessment.	None
Bryer et al. The South African guideline (33) (Stroke)	2011	Early assessment and planning of discharge and comprehensive assessment of medical problems, impairments and disabilities by specialist staff is needed.	None
Swedish National Board of_Health and Welfare. Quality and efficiency of stroke care in Sweden (55). (Stroke)	2011	No recommendations for OM.	None recommended
Venketasubramanian et al. Singapore Clinical Practice Guidelines Workgroup on Stroke (56) (Stroke)	2011	Recommends multi-disciplinary medical assessment in acute stroke or transient ischemic attack (TIA). No reference to UE assessment	None
Guideline 118. SIGN. Management of patients with stroke (57). (Stroke)	2011	Assessment of patient's needs to set goals and re-assess progress against goals. No reference to UE assessment	None
Estonian clinical guidelines for stroke rehabilitation (27) (Stroke)	2011	Use of valid and standardized measures including assessment of sensorimotor function, cognition, speech, and ADL in predefined time points.	NIHSS (51), FIM (53), Barthel Index (50), Modified Ashworth Scale (58); Berg Balance Test (59)
New Zealand Clinical guidelines for Stroke Management (60). (Stroke)	2010	Reference to assessment in acute care and of those who want to return to work.	None

The Dutch National guideline (17), provided very comprehensive recommendations on the diagnostic process and included recommendations for specific tools, within each ICF domain, that should be used for diagnosis—to allow informed clinical decision-making; to predict recovery and to assess progress. Recommendations are summarized as follows: Any patient with a stroke should be systematically assessed in terms of body functions, activities, and participation prior to the start of the physical therapy process, preferably using reliable, valid, and responsive measurement instruments. These measurements should be administered at predefined moments during the physical therapy process, in order to objectively monitor the patient's clinical course. Basic upper limb measurement should include: muscle strength, dexterity and ADL. Tools were selected by the guideline development team on the basis of their reliability, responsiveness, predictive and construct validity, and finally their practical feasibility. They make recommendation for future practice: “*many publications fail to report follow-up data, and if they do, the timing of follow-up assessments varies widely. This means that the long-term added value of nearly all interventions is unknown*.” It is suggested that “*frequent and systematic assessment of functional changes over time (monitoring)”* is an important factor contributing to higher quality of care. They recommend considering measures before each multidisciplinary meeting.

The US National Guideline (14) also makes comprehensive recommendations on assessment for best clinical practice. It acknowledges the need for a single assessment used throughout the course of stroke recovery, referring to measures of body function/structure and citing the upper limb motor section of the Fugl-Meyer scale or the Box and Block Test for measuring arm motor deficits. The Australian Guideline (38), focuses on interventions, but recommends assessment using valid measures, although without reference to physical assessment of the upper limb. The New Zealand (60) guideline makes recommendations on all aspects of stroke management and prevention based on level of evidence, expert opinion and clinical experience, however, the only reference to assessment is in relation to acute care and of people who want to return to work.

Six UK Guidelines (of which two were Scottish) were found: three for Stroke (16, 28, 61), one for SCI (54), one for brain injury (62), and one for MS (32). The Royal College of Physicians (RCP) stroke Guideline is a comprehensive guideline for best clinical practice. The RCP Guideline considered the general principles of measurement in stroke rehabilitation, for example the importance of measuring function and understanding which domain of the WHO ICF framework an instrument is measuring. It states that instruments should be appropriate to the intervention in question and clinicians should be trained in the use of measurement scales to ensure consistent use within the team. The National Institute of Clinical Excellence (NICE) recommendations (28) guidelines were mainly concerned with the organization of health and social care and specifically the delivery of best practice. Specific recommendations were: screening on admission and on transfer from hospital to community using the WHO ICF to provide information on functional abilities; use of standardized screening instruments; treatment and assessment should be provided by HCPs who have appropriate skills and training and patients should be assessed and fitted for wrist and hand splints by trained HCPs. The third UK guideline on MS makes no reference to upper limb problems, however does specify that assessments should be conducted by a “healthcare professional with appropriate expertise in rehabilitation and MS.” The fourth UK Guideline, on SCI (63) also makes no reference to upper limb assessment, focusing only on medical assessment except for brief reference to the need for a musculoskeletal assessment including spasticity, joint range of movement, and pain. Neither the Singapore (56) nor the Swedish (55) Guidelines make recommendations on assessment. The Singapore Guidelines ^1^(56) state the importance of assessment in acute stroke, giving recommendations, but make no reference to assessment in rehabilitation. Although not an official National publication, we have included the Canadian Web-based Stroke Rehabilitation Evidence-Based Review SREBR guidelines^1^ which provide comprehensive recommendations on assessment and present level of evidence for a wide range of clinical scales. The SREBR consolidates the best available scientific evidence for the effectiveness of stroke rehabilitation and is an excellent resource. The review is constantly updated and includes a substantial section on OMs. The SREBR used the ICF Framework and in addition to the usual measures of reliability and validity, also considered appropriateness and responsiveness (floor and ceiling effects), precision, interpretability, acceptability, feasibility, and the thoroughness of testing. The scope is very wide, including tests for cognition, depression etc. It does not address upper limb assessments *per se*, but includes a number of UL focussed impairment and activity measures, which are scored in each category.

Nineteen other articles were included in the review (Table 2). Peer review articles were generally less comprehensive than the National Guidelines and often focused on a specific area of neurological rehabilitation, for example Occupational Therapy or tele-rehabilitation. They were however more focused on upper limb OMs and some gave recommendations for specific measures.

**Table 2:** Summary of the peer reviewed and practice guideline records included in the review.

**Record**	**Year**	**Summary of recommendations**	**Recommended measures**
Wechesler et al. (23) (Stroke)	2017	Improve quality monitoring and outcomes and consider sharing patient data. NIHSS score done remotely during transit to hospital (64)	NIHSS score (51)
Intitut National d'excellence en sante et en sociaux—(TBI)	2017	Guidance on global assessment and rehabilitation interventions including motor control. No specific reference to, or recommendation for UE assessment	None
ATAXIA UK. *Ataxia UK* (24) (Non-specific pathology)	2016	No reference to UE specifically. Measure patient engagement and satisfaction with the performance of an activity,	Assessment of Motor and Process Skills (AMPS) (65), Goal Attainment Scale (GAS) (66), Canadian Occupational Performance Measure (COPM) (52), self-efficacy tools and quality of life measures.
Wolf et al. (67) (Stroke)	2015	No recommendations for assessment	None
Hebert et al. Canadian stroke best practice recommendations (37) (Stroke)	2015	Assessment within 48 h including: function, safety, physical readiness, and ability to learn and participate in rehabilitation. No specific reference to UE	None
Majersik et al. (25) (Stroke)	2015	Studies exploring genetic factors should also measure stroke outcomes. Medical and global outcomes, impairment and activity early post stroke, at 3 months and ideally at 6 and 12-months' post stroke. Document access to and amount of therapy	No specific upper limb measures. NIHSS (51), GAS (66), FIM (53)
Haselkorn (68) (Stroke)	2015	No specific recommendations	None
College of Occupational Therapists and Association Of Chartered Physiotherapists in Neurology. (15). (Splinting. Non-specific pathology)	2015	Use valid and reliable measures across the ICF framework. Global measures are unlikely to be sensitive to changes, but should be included; choice and timing of OM is important. Recommendations for future research include use, choice and timing of OM	Arm activity measure (69) Visual analog scale (70); ARAT (46)
Potter et al. (71). (MS)	2014	Important to consider measures that can be used in different settings (hospital vs. home) to track patients over a long period	No specific recommendations
Billinger et al. (72). (Stroke)	2014	No specific OM for UL	None
Finlay and Evans (metastatic spinal cord compression). (21) (SCI)	2014	Pain, motor and sensory dysfunction assessment should be carried out within 24–48 h of admission and prior to discharge. Pain should be re-assessed at least daily. Only when the MSCC is deemed stable or more active rehab is permitted can the full assessment be completed. A wide range of measures can be obtained through: http://www.rehabmeasures.org/default.aspx	Light touch sensation; Sharp/blunt or pin prick sensation; Joint proprioception; Muscle power (myotome chart and Oxford classification); Muscle tone: flaccidity or spasticity (MAS) (58); Joint ROM (active/passive) and muscle length; Personal activities of Daily Living (PADL): Activities of Daily Living (ADL):
Ontaneda et al. (MS) (73). (MS)	2012	A universally accepted measurement instrument that is precise, reliable, easy to administer, captures key neurological domains affected by MS, is sensitive at all levels of disability and accurately reflects neurological and neuropsychological disability is still lacking. Agreeing on single clinical measure that is useful at all stages of the disease is challenging	Multiple Sclerosis Functional Composite (MSFC) approach. Recommends the development of a database focused on MSFC and follow-up projects aimed at developing patient-reported outcomes, imaging markers, and biological markers of the MS disease process.
Canadian EBRSR (http://www.ebrsr.com/) (18) (Stroke)	2012	Use of the ICF Framework; reference to reliability, validity, appropriateness and responsiveness (floor and ceiling effects), precision, interpretability, acceptability, feasibility. Does not address UE assessments per se, but includes a number of UE focussed impairment and activity measures, which are scored in each category. Provides information for selection of most appropriate measure.	Impairment: FMA (69), and MAS (47, 74) Activity: ARAT (46), B&B (43), Chedoke-McMaster (75), FIM (53), 9HPT (43, 76), WMFT (77) Participation: COPM (52)
Miller et al. (34). (Stroke)	2010	Hypertonicity should be assessed, but no recommended tools. The MAS has poor validity and inter-rater reliability. Other measures have not been shown to be feasible clinically. Acknowledges importance of trained assessors. Recommends ADL Assessment post-discharge from rehabilitation Tools should be agreed by the MDT and be valid and reliable. No reference to UE	15 Upper Limb Motor assessments are listed as ‘commonly used’
Hachinski et al. (35). (Stroke)	2010	Calls for consensus on, then implementation of, standardized clinical and surrogate assessments. No reference to UL Tools for measuring the biology of stroke recovery are needed to inform optimal timing, intensity, duration, and content of therapy. The best standardized measures of behavior and outcomes after stroke need to be defined and used in clinical practice. Standardized rater training needs to be developed. Surrogate markers of treatment effect could also be used as predictive tools for outcome and thus be of value for entry criteria in clinical trials or in evaluating treatment outcomes and guide clinical decision-making. No specific reference to UL assessment.	None
VA/DOD The Management of Stroke Rehabilitation (22) (Stroke)	2010	NIHSS performed by trained, certified assessors within the first 24 h, and consider re-assessing prior to discharge from acute care. Motor function assessed at impairment and activity levels using assessments with established psychometric properties. A standardized assessment tool should be used to assess ADL/IADL A MDT assessment should be undertaken to establish the patient's rehabilitation needs and goals.	Functional Independence Measure (FIM) (53). NIHSS (51) Motor function: muscle strength for all muscle groups, active and passive range of motion, muscle tone, ability to isolate the movements of one joint from another, gross and fine motor co-ordination. The daily use of the paretic extremity should be assessed using a self-report measure (e.g., the Motor Activity Log) (47) and accelerometery.
Alexander et al. (78) (SCI):	2009	Evaluation of UE impairment is important, but generic tests of hand function are ill-suited for use with persons with SCI, with the exception of the Grasp and Release test - developed to assess the effect of a neuroprosthesis.	Grasp and release test (79)
Gall et al. (63) (SCI).	2008	No reference to upper limb assessment, except for brief general mention of spasticity, joint range of movement, and pain assessment	None
Steeves et al. (80). (SCI)	2007	Recommends assessment of UE function, including sensation in clinical trials and acknowledges lack of agreement and absence of SCI specific tests for SCI and lack of sensitivity in current measures. Discusses a range of tools without giving specific recommendations	Accurate sensitive and functional measures
Bayley et al., ABIKUS (36) (TBI).	2007	Recommendations based on a systematic review. Recommends assessment of spasticity and motor function by trained professionals	None

In total, 51 outcome measures were recommended, of which 39 addressed stroke (76%), 5 TBI (10%), 3 SCI (6%), 1 MS (2%). Four outcome measures (8%) were recommended without specifying which pathology it should be used for. Regarding stroke guidelines, the most frequently recommended OMs were NIHSS (5), FIM (4), Barthel Index (3), and FMA (3). For the other pathologies, recommended OMs were scattered across different OMs.

We have synthesized recommendations made by the National Guidelines and published articles under the following headings: Why, when and by whom assessments should be conducted and what should be measured.

#### Why Assessment Is Important

“Not Everything That Counts Can Be Counted” (81) but without valid, reliable and sensitive measures that are meaningful to patients, clinicians and researchers our field cannot advance. We will not know what works, when or with whom. Neurological rehabilitation is complex in terms of both patients and intervention (26, 57) There are few interventions or conditions for which there is a single measure as there is for example in testing a new drug for hypertension. Winstein (14) acknowledges the challenge faced in assessing services, patient outcomes and effectiveness of neurological rehabilitation stating that: “*the array of rehabilitation services delivered to stroke patients in the United States is broad and highly heterogeneous, varying in the type of care settings used; in the duration, intensity, and type of interventions delivered.”* and that this “*brings with it challenges in terms of determining the quality of care delivered by the system”* and “*in terms of assessment of which research findings…are applicable to the system.”* Alexander (78) identified the need for agreed measures in their multi-disciplinary study of current and evolving tools for evaluating people with spinal cord Injury (SCI), reporting that none of the findings of major clinical trials of new interventions had translated into standard care and argued that to achieve translation, “*agreed, appropriate and valid primary end points and intervention protocols are needed.”*

#### When During the Rehabilitation Period Should Assessments be Conducted?

Nine publications (seven stroke) referred to timing of assessments in relation to rehabilitation recommending soon after admission and on transfer of care. Beyond that there was wide variation, particularly in frequency of assessments. The Dutch Guidelines recommended that patients were assessed within 1 week of admission and discharge (or when transferring treatment to another colleague) and at the end of the 1st week, 3rd and 6th month post-stroke. They also recommended considering measures before each multidisciplinary meeting. The NZ guidelines stated that patients should be assessed when treatment choices were being made, as assessments were fundamental to measuring deficits, planning goals, and planning management. It recommended that all assessments occurred as soon as possible after admission (aiming for within the first 2 days) with the stroke team working together so as not to overburden the patient by duplicating questions.

The COT and ACPIN Report (82) was concerned with splinting and suggested that specified outcomes should be recorded at baseline and at defined intervals, but they did not suggest what these should be (25). Winstein (14), recommends that “*all patients should undergo a formal assessment of their rehabilitation needs before discharge”* and Finlay (21) recommend that physiotherapy assessments be carried out within 24–48 h of admission and that the assessment should include pre-admission mobility and motor dysfunction. The Canadian best practice guidelines state initial screening and assessment should be conducted within 48 h by rehabilitation professionals.

There were only two publications which referenced timing of assessment in MS and SCI, The American Physical Therapy Association Neurology Section task force recommended using OM to track MS patient status over a long-term period or as patients transition across settings (71). The Guidelines and Audit Implementation Network (GAIN) recommends PT and OT therapy assessments (pain, motor and sensory dysfunction) for SCI should be carried out within 24–48 h of admission and prior to discharge.

#### Clinical Utility—Who Should Conduct the Assessment

A strong consensus was found in favor of assessments being conducted by appropriately trained HCPs. Patients with difficulties in performance of daily activities should be assessed by a clinician trained in the use of whichever scales are chosen to ensure consistency of their use within the team and an understanding of their purposes and limitations (60). This view is supported by (34) recommending that clinicians obtain not only training to establish administration and scoring consistency, but also, routine retraining to ensure they maintain this consistency (71). They highlight the fact that although OMs have benefits in physical therapist practice multiple barriers interfere with their use, most notably, a limited understanding of how to select and apply the best OM.

#### Single vs. Multiple and Specific OMs, Within the ICF Framework

No records recommended a single OM with the exception of Winstein (14) who suggested the use of a computerized questionnaire called the “*Activity Measure for Post-Acute Care”* as an outcome measure for all stroke patients to “*track stroke rehabilitation outcome.”* Billinger (72) suggested that accelerometery was likely to be used as an OM for future clinical trials as it measured changes in free-living physical activity and compliance with exercise programmes.

There was consensus between the Dutch, UK, and US guidelines that patients should be assessed in each domain of the ICF framework, but conflict between using a single measure to enable progress to be monitored throughout recovery and multiple measures to allow for changes in setting, goals and ability levels. The US guidelines recommend multiple OMs whereas the most recent stroke guidelines from the UK National Institute for Health and Care Excellence (NICE) (28) recommend primary and secondary OMs, with the primary assessing function and secondary including measures of impairment, activity limitation and quality of life. The Scottish Intercollegiate Guidelines Network (26) recommended using a range of assessment tools to assist goal-setting. Multiple OMs were often recommended (14, 15, 17, 21, 71) arguing, for example, that it would be challenging to select only 1 or 2 OMs for use with all people with Motor Neurone Disease (MD) and Multiple Sclerosis (MS) (83, 84) due to variation in disability levels and treatment in a variety of settings. Ontaneda (73) concurred, recommending different OMs for people at different stages of MS and the RCOT (85) agreed with (71) that a “*one size fits all”* intervention with a single outcome measure was of limited, if any, value. The SIGN TBI guideline (26) stated that because rehabilitation interventions usually target multiple or complex outcomes, and because individual goals vary, a single measure may be impossible or inappropriate.

#### Assessment of Body Function and Structures (Impairment)

The US Guidelines were skeptical about the use of measures in the body structure and function (impairment) domain of the ICF framework, considering that the psychometric properties of tools had not been established. They referred specifically to measures of spasticity/hypertonicity citing the equivocal evidence for validity and inter-rater reliability for the Modified Ashworth Scale. The VA/DOD Guidelines (22) however, made very strong and clear recommendations for measuring motor function both at the impairment (ability to move in a coordinated manner in designated patterns) and at the activity level (performance in real life or simulated real life tasks) using assessments with established psychometric properties.

In terms of measuring spasticity, Miller et al. (34) acknowledged the problem of validity and interrater reliability of the most commonly used Modified Ashworth Scale, but that other spasticity measures reported in the literature have problems with respect to clinical feasibility and the range of joints that could be assessed. Alexander (78) was one of the few to discuss the use of electrophysiological measurements such as Electromyography (EMG), Motor Evoked Potentials (MEPs) and Somatosensory Evoked Potentials (SEPs) to assess spinal conductivity and spasticity in SCI. Hachinski (35) was one of the few records to refer to the need for assessments to measure the mechanisms of recovery. It reported the consensus of a “Synergium,” commissioned to finding new ways of accelerating progress in reducing the risks, effects, and consequences of stroke.

#### Assessment of Activities of Daily Living (ADL) and Participation

While upper limb function has a significant impact on ADL, QoL and participation, it is beyond the scope of this review to consider in detail the recommendations for OMs in these categories, especially as they do not specifically assess the upper limb. The Dutch guidelines, however, proposed a range of measures to assess factors that may impact on recovery of UL function and therefore ability to participate in everyday life (17).

#### Psychometric Properties and Appropriateness of OMs

The Australian Guidelines recommended that Clinicians use tools that meet the needs of the patient and are valid and reliable in the stroke population. The NZ guidelines added that while, because of the enormous variety of assessment tools and measures, they did not make specific recommendations, it was important to choose a specific tool based on the validity (in a stroke population), reliability, and availability. Miller (34) recommended standardized, valid and reliable test procedures to document the severity of upper and lower limb impairment and to document the levels of assistance needed for mobility. Alexander (78) emphasized the importance of using measures that were valid, reliable and sensitive in the SCI population and concluded that further work was needed on existing measures to identify the most appropriate tools for specific targets. Finlay (21) directed the reader to The Rehabilitation Measures Databases^2^ both of which provide information on a wide range of useful assessments and OMs. These are excellent repositories of measures, providing information on conditions where they might be used, availability, time taken to complete the tests, training required to conduct them and links to references, some of which include data on psychometric properties. They do not, however, make recommendations *per se*.

#### Self-Efficacy and Goal Orientated Measures—Assessment Integrated Into Therapy

The RCP (16) recommended that people with stroke should be helped to identify goals with specific, time-bound and measurable outcomes, but does not recommend specific measurement tools to assess whether goals have been achieved. There is a clear distinction between measuring what a person “can do” and what they “do do.” Many of the standardized, recommended and commonly used measures of impairment and activity do not address the latter, whereas Patient Reported Outcome Measures (PROMs) and measures of self-efficacy, focus on what the patient actually does (or reports doing) in their day-to-day life. In relation to this, Ataxia UK (24), stated that OMs should focus on engagement and satisfaction because a tool that measures impairment does not always demonstrate effectiveness. The Management of Stroke Rehabilitation Report (22) recommended both a self-report measure (e.g., the Motor Activity Log) and an objective measure (e.g., accelerometry) to assess daily use of the affected upper limb and also as a motivational or self-management tool for participants taking part in clinical trials (72). Despite these recommendations, the review of OMs used in (neurorehabilitation) limb splinting evaluation studies, conducted by the Royal College of Occupational Therapy (RCOT) and Association of Physiotherapists in Neurology (ACPIN), found that patient satisfaction was the least common OM used (82).

### Risk of Bias

Data sources were predominantly English language, which may have biased the main findings. However, in mitigation, as authors, who were members of the COST Action, covered several languages we were able to search for (and include) National Stroke Guidelines in a range of languages. Differences in health care systems worldwide may also have been a source of bias reflected in the recommendations made in the primary publications.

Finally, the quality of identified guidelines was not evaluated with a standard tool such as AGREE II (Appraisal of Guidelines for REsearch and Evaluation). AGREE generates summary scores, in which all items and domains have equal weight. This tool is useful in judging the quality of the Guidelines and was used in Jolliffe et al.'s recent systematic review of Clinical Guidelines for Stroke and other Acquired Brain Injuries (86). However, their aim was to identify high quality guidelines, whereas ours was more specific; to “identify what recommendations are made for upper limb assessment.” Instead we therefore used descriptive analysis to identify evidence-based consensus on upper limb assessment across multiple pathologies to generate an in-depth knowledge of the quality and content of each guideline.

## Discussion

### Summary of Main Findings

Our review of National Guidelines and published articles on recommendations for OMs in UL rehabilitation following Stroke, MS, SCI, and other neurological conditions has identified some areas in which there is a clear consensus. For example, that assessment is important in neurological rehabilitation, should encompass all domains of ICF Framework and that, with one exception, multiple OMs should be used. Where recommendations included protocols for use of OMs, there was no disagreement to the following: they should be applied by HCPs who are trained to use them and at regular intervals during the rehabilitation pathway. Although intervals vary, global measures are recommended within 24 h of admission and UL specific measures within 1 week. All published articles and Guidelines recommend early assessment and assessment prior to discharge, while many recommend far more frequent assessments. The importance of linking assessment to goal-setting (24, 57, 61), the use of measures to encourage and motivate patients (24) as well as the importance of patient reported outcome measures (PROMS) (22) was evident. These recommendations reflected recognition of the importance of self-efficacy and independence and PROMS to assess what a patient actually does rather than can do is important. What we found lacking was recommendation to use specific outcome measures for which validity and reliability have been demonstrated. There was also lack of consensus on which measures should be used; although there was more agreement about global measures of participation and ADL than UL specific measures of impairment and activity limitation. The FIM for example is recommended in six reviews.

There was very little agreement across the Guidelines about what outcome measures should be used, even within pathologies and the categories of the ICF (Table 3). Even regarding the condition for which the majority of OM recommendations were made (76%), stroke, guidelines fail to agree on a specific set of OMs to be used. The most frequently recommended OMs in stroke guidelines were three global stroke OMs (NIHSS, FIM, Barthel Index) and only 1 specific upper limb OM (FMA). Two of those regarded OMs on Activity level (global), NIHSS, and FIM, between which no consensus was apparent either.

**Table 3:** Frequency with which different outcome measures were recommended in total and for each pathology included in the review.

**Domain**	**Outcome measures**	**Total number of records/References**	**Number of records per pathology**				
			**Stroke**	**MS**	**SCI**	**TBI**	**Other**
Impairment	Fugl-Mayer Assessment (FMA)	3 (14, 17, 18)	3	0	0	0	0
	Modified Ashworth Scale (MAS)	2 (17, 21)	1	0	1	0	0
	Muscle power (Myotome chart and Oxford grading)	1 (21)	0	0	1	0	0
	Passive Range of motion	2 (21, 22)	1	0	1	0	0
	Electro-goniometer (range of motion)	1 (14)	1	0	0	0	0
	Grip strength (e.g. Jamar dynamometer)	1 (14)	1	0	0	0	0
	Co-ordination and selective muscle activity	1 (22)	1	0	0	0	0
	Grasp and release test	1 (78)	0	0	1	0	0
	Box and Block test (BBT)	1 (14)	1	0	0	0	0
	Nine-hole-peg-test (9HPT)	2 (17, 18)	2	0	0	0	0
	Motricity Index (MI)	1 (17)	1	0	0	0	0
Impairment (Sensation and Pain)	Visual Analog Scale (VAS)	1 (15)	1	0	0	0	0
	Light touch	1 (21)	0	0	1	0	0
	von-Frey filaments	1 (14)	1	0	0	0	0
	Proprioception	1 (21)	0	0	1	0	0
Activity (UL)	Wolf Motor Function Test (WMFT)	1 (18)	1	0	0	0	0
	Assessment of Motor Processes and Skills (AMPS)	1 (24)	0	0	0	0	1
	Arm Activity Measure	1 (15)	0	0	0	0	1
	Action Research Arm Test (ARAT)	3 (15, 17, 18)	2	0	0	0	1
	Chedoke McMaster	1 (18)	1	0	0	0	0
	Computerized questionnaire	1 (14)	1	0	0	0	0
	Frenchay Arm test (FAT)	1 (17)	1	0	0	0	0
Activity (Global)	National Institute of Health Stroke Scale (NIHSS)	5 (17, 22, 25, 27, 28)	5	0	0	0	0
	Canadian Occupational Performance Measure (COPM)	1 (62)	1	0	0	1	0
	Goal Attainment Scale (GAS)	2 (24, 25)	1	0	0	0	1
	Functional Independence Measure (FIM)	5 (18, 22, 25–27)	4	0	0	1	0
	Multiple Sclerosis Functional Composite (MSFC)	1 (73)	0	1	0	0	0
	Motor Activity Log (MAL)	1 (22)	1	0	0	0	0
	Berg Balance Scale (BBS)	1 (27)	1	0	0	0	0
Participation and QoL	Barthel Index (BI)	4 (17, 26–28)	3	0	0	1	0
	Personal Activities of Daily Living (PADL)	1 (21)	0	0	0	1	0
	Nottingham Extended ADL	1 (17)	1	0	0	0	0
	Stroke Specific Quality of Life Scale (SSQoL)	1 (17)	1	0	0	0	0
	Total = 52		39	1	3	5	4

Without an internationally agreed core set of outcome measures that satisfy the requirements identified in this review, progress in neurorehabilitation will remain hampered and data will be wasted. From the research perspective, it is well-known that clinical trials of conventional and novel interventions are expensive, often return equivocal results and frequently fail to recruit adequate samples of patients. An important way that we can advance the field of neurorehabilitation, gain a better understanding of the recovery processes and disease progression and understand what works, with whom, when and in what dose is through meta-analysis of multiple trials, audits and longitudinal studies. Meta-analysis can only be done effectively if common outcome measures have been applied. Lack of meta-analyses impacts not only research into effectiveness of existing and novel therapies but also in delivering best practice.

National strategies and frameworks continue to emphasize the need for informed decision making in healthcare that are research led and evidence-based, yet the UK, Australian and US National Clinical Guidelines for Stroke indicate that there is limited research to assess efficacy of rehabilitation technologies, either individually or in combination (14, 16, 31).

### Limitations

This systematic review has explored “National Guidelines,” or “practice guidelines,” and “recommendations” published in peer-reviewed journals, focusing on assessment of the UL. We did not generate quantitative data, conduct a statistical analysis or use a standardized tool to assess the quality of the publications (see section on risk Bias above). We included all guidelines that satisfied our criteria and have not provided critical analysis of the quality of each publication.

## Conclusion

Clinical practice guidelines provide very little specific guidance on assessment of the UL, even within ICF domains and/or pathology-specific recommendations. Agreement on a core set of OMs is not achieved by systematic reviews of guidelines such as this, predominantly due to a lack of explicit OM recommendations in most of the identified guides. Nevertheless, our extensive and rigorous review has provided a comprehensive summary of current recommendations, and therefore arguably current use of OMs. Defining a core set of measures and agreed protocols requires international consensus between experts representing the diverse and multi-disciplinary field of neurorehabilitation. The group should include representation from research and clinical practitioners as well as rehabilitation technology researchers and commercial developers, so that recommendations are made cognoscente of the future potential for technology in assessment and neurorehabilitation. If such a consensus was achieved, a standardized approach to assessment would make research findings more meaningful and provide a benchmark for quality in clinical practice and potentially improved standards and more cost-effective neurorehabilitation. Our review has identified agreement that assessment is critical and should encompass body function and structure, activity and participation and that there is a need for standardized measures.

## Author Contributions

JanB led the project and was the main author of the manuscript. All authors contributed to conception, protocol, and design of study and to acquisition of data. Critical revision of the report for important intellectual content. JanB and AH conducted the initial literature search and LM conducted an updated search. JanB, AH, and LM screened records with input from all other authors where needed.

### Conflict of Interest Statement

TK was employed by Fundation Tecnalia Research and Innovation. The remaining authors declare that the research was conducted in the absence of any commercial or financial relationships that could be construed as a potential conflict of interest.

## Footnotes

^1^(http://www.ebrsr.com/)

^2^http://www.rehabmeasures.org/default.aspx and http://www.neuropt.org/professional-resources/neurology-section-outcome-measures-recommendations
